# WISP1 mediates lung injury following hepatic ischemia reperfusion dependent on TLR4 in mice

**DOI:** 10.1186/s12890-018-0744-z

**Published:** 2018-12-06

**Authors:** Yao Tong, Zhuang Yu, Renlingzi Zhang, Xibing Ding, Zhixia Chen, Quan Li

**Affiliations:** Department of Anesthesiology, Shanghai East Hospital, School of Medicine, Tongji University, 150 Jimo Road, Shanghai, 200120 China

**Keywords:** Hepatic ischemia-reperfusion injury, WNT1 inducible signaling pathway protein 1, Inflammatory cascades, Toll like receptor

## Abstract

**Background:**

Hepatic ischemia-reperfusion injury (IRI) is a common pathological phenomenon, which causes hepatic injury as well as remote organ injuries such as the lung. Several mediators, such as oxidative stress, Ca^2+^ overload and neutrophil infiltration, have been implied in the pathogenesis of liver and remote organ injuries following reperfusion. WNT1 inducible signaling pathway protein 1 (WISP1) is an extracellular matrix protein that has been associated with the onset of several malignant diseases. Previous work in our group has demonstrated WISP1 is upregulated and contributes to proinflammatory cascades in hepatic IRI. However, the role of WISP1 in the pathogenesis of lung injury after hepatic IRI still remains unknown.

**Methods:**

Male C57BL/6 mice were used to examine the expression and role of WISP1 in the pathogenesis of lung injuries after hepatic IRI and explore its potential mechanisms in mediating lung injuries.

**Results:**

We found WISP1 was upregulated in lung tissues following hepatic IRI. Treatment with anti-WISP1 antibody ameliorated lung injuries with alteration of cytokine profiles. Administration with rWISP1 aggravated lung injuries following hepatic IRI through excessive production of proinflammatory cytokines and inhibition of anti-inflammatory cytokines.

**Conclusions:**

In this study, we concluded that WISP1 contributed to lung injuries following hepatic IRI through TLR4 pathway.

## Introduction

Hepatic ischemia-reperfusion injury (IRI) is a pathological phenomenon event when hypoxic liver undergoes oxygenic blood reperfusion. Usually IRI can be divided into two categories, namely warm ischemia which often occurs in trauma, shock, and liver transplantation with temporary blood interruption and cold ischemia which appears during organ preservation before transplantation. Recent studies have implied several pathological mechanisms in the pathogenesis of IRI including production of reactive oxygen species (ROS), synthesis of inducible nitric oxide synthase (iNOS), and secretion of proinflammatory cytokines and chemokines which leads to immune cell (especially neutrophil) recruitment and inflammatory cascades [1–3]. Excessive production of proinflammatory cytokines in the serum, including TNF-α, IL-1β and IL-6 in the early phase contribute to the local and remote organ damage [4, 5]. Previous studies have confirmed lung injury in the IRI model [6]. However, the exact mechanism of IRI induced remote organ injury still remains unclear.

Pattern recognition receptors (PRRs) have been demonstrated to participate in the pathogenesis of IRI, with one of the most important members being Toll-like receptor 4 (TLR4) [7]. TLR4 can recognize various kinds of endogenous antigens and is activated in IRI in liver and remote organs [8]. The functions of TLR4 on immune cells are more vital than on hepatocytes although the regulating mechanisms may be different [9, 10]. Besides TLR4 signaling, other PRRs, such as TLR2 and TLR9, have been reported to be involved in the pathogenesis of IRI [11–13].

WNT1 inducible signaling pathway protein 1 (WISP1) is a secreted extracellular matrix (ECM) protein which is ubiquitously expressed in multiple organs, such as lung, liver, kidney, heart and small intestine [14]. WISP1 belongs to the CCN family which contains 6 members, namely CCN1 (cysteine-rich protein 61, Cyr61), CCN2 (connective tissue growth factor, CTGF), CCN3 (nephroblastoma overexpressed gene, NOV), CCN4 (WNT1 inducible signaling pathway protein-1, WISP1), CCN5 (WISP2) and CCN6 (WISP3) [15]. The functions of WISP1 has been linked to cell proliferation, survival and differentiation [15]. Recent studies have demonstrated that WISP1 relative expression is significantly upregulated in some diseases, including lung carcinoma, hepatocellular carcinoma [16] and colon adenocarcinomas [17]. Interestingly, Li et al. have linked WISP1 to the progression of inflammation [18], that is, stimulation of recombinant WISP1 aggravates proinflammatory responses in LPS stimulated macrophages. Previous work in our group has demonstrated WISP1 is upregulated and contributes to proinflammatory cascades in hepatic IRI [19]. However, the role of WISP1 in the pathogenesis of lung injury after IRI still remains unknown.

In this study, we aimed to investigate the WISP1 expression in the lung tissue after hepatic IRI and determine the regulating mechanisms and functions of WISP1 in the lung injury after hepatic IRI.

## Methods

### Mice

C57BL/6 mice were purchased from Shanghai Laboratory Animal Co Ltd. (SLAC, Shanghai, China). TLR4 knockout (TLR4 KO) mice were kindly provided by Dr. Timothy R. Billiar (University of Pittsburgh, USA). All mice were raised in specific pathogen-free condition. Male mice of 8–10 weeks (weight 18.4.8 ± 1.8 g) old were used for experiments. Animal experiments were authorized by the Ethics Committee of Tongji University.

### Induction of hepatic I/R injury model]

The induction of segmental (70%) hepatic hepatic warm I/R injury model was performed as previously described with minor modifications [8, 20, 21]. Mice were treated with isotype control IgG or neutralizing WISP1/WISP2 antibody (MyBioSource, 6 μg/g) intraperitoneally 1 h before ischemia and again at the time of reperfusion. In another experiment, mice were administered with recombinant WISP1 protein (rWISP1, 1 μg/g) or sterile phosphate-buffered saline (PBS, w/o Ca^2+^/Mg^2+^) intraperitoneally immediately after reperfusion. Sham mice were sufficiently anesthetized, and then a midline abdominal incision was made. The portal triad was exposed without further treatment for liver ischemia. Mice were sacrificed at the predetermined time points (0 h, 3 h, 6 h, 12 h and 24 h) after reperfusion for collecting serum and lung tissues.

### Lung edema measurement

Lung edema was evaluated as previously reported by an increase in the wet-to-dry (W/D) weight ratio of the lungs [21, 22]. The left lung was dissected and weighted before and after drying in a micro oven at 65 °C for 48 h. W/D ratio was then detected.

### Alveolar-capillary permeability assay

Evans blue albumin (EBA) was used to measure Alveolar-capillary permeability as previous illustrated [23]. Briefly, the internal jugular vein of mice was injected with EBA (25 mg/kg) 1 h before sacrificed. And then, the right lung and blood samples were available for further steps.

### Evaluation of Bronchoalveolar lavage fluid (BALF)

BALF was evaluated as previously publications in our group [21]. Mice were instilled with 1 mL PBS and approximately 80% fluid was retrieved. BALF was kept on ice immediately after recovered and was then centrifuged at 1000×g, 4 °C for 5 min. Supernatants were used for total protein concentration examination and cytokine levels, which can keep at − 80 °C for long-term preservation. Total cell counts were determined using a hemocytometer.

### RNA extraction, reverse transcription PCR, Quantative real-time PCR

Total RNA was extracted from lung tissues using TRIzol reagent (Sigma-Aldrich) according to established protocols in our group [21, 24]. The total concentration of RNA was measured at 260 nm with a spectrophotometer (BeckmanCoulter, Brea, CA, USA). First strand complementary DNA (cDNA) synthesis was performed using PrimerScript RT Master Mix (Takara Bio Inc., Shiga, Japan) according to the manufacturer’s protocols. Real-time PCR for glyceraldehyde-3-phosphate dehydrogenase (GAPDH), WISP1, IL-6, TNF-α, IL-10 was performed using SYBR premix Ex Taq™ (Takara) with a Step One Plus real-time PCR system (Applied biosystems, USA), under the manufacturer’s instructions. GAPDH was used as house-keeping gene to normalize the gene expression. Gene relative expression was calculated using the 2^-ΔΔ^Ct method. All of the primers were synthesized by Sangon Biotech (Shanghai, China). Primers sequence was as follows: mWISP1 forward 5′- CGCCCGAGGTACGCAATAG, reverse 5′- GCAGTTGGGTTGGAAGGACT, mIL-6 forward 5′- CTGCAAGAGACTTCCATCCAG, reverse 5′- AGTGGTATAGACAGGTCTGTTGG, mTNF-α forward 5′- CAGGCGGTGCCTATGTCTC, reverse 5′- CGATCACCCCGAAGTTCAGTAG, mIL-10 forward 5′- CTTACTGACTGGCATGAGGATCA, reverse 5′- GCAGCTCTAGGAGCATGTGG.

### Enzyme-linked immunosorbent assay (ELISA)

BALF supernatants were analyzed for mIL-6, mTNF-α and mIL-10 cytokines using a sensitive commercial ELISA kit (R&D Systems) according to the manufacturer’s instructions.

### Western blot analysis

Western blot analysis was performed as previously described in our studies [21, 24]. Lung tissues were dissected and lysed using iced-cold lysis buffer (Beyotime, catalog no. P0013G). Protein concentration was determined via standard BCA assay. After gel electrophoresis, protein was then transferred to a nitrocellulose membrane and blocked using 5% nonfat milk for 1 h. Membranes were incubated with rabbit anti-mouse WISP1 antibody (1:400, R&D systems) and β-actin antibody (1:10000, Proteintech) at 4 °C overnight, and then incubated with secondary antibody for 1 h at 37 °C. Odyssey image analysis system (Licor Biosciences) was used to quantify.

### Immunofluorescence

Immunofluorescent staining was performed using standard protocols [25, 26]. Briefly, 4–6-mm frozen sections from murine lung tissues were incubated with anti-WISP1 antibody (abcom, 1:200) antibody overnight at 4 °C. Sections were then incubated with secondary Alexa Fluor 488-labelled rabbit anti-mouse antibody (CST, 1:200) for 1 h at room temperature. Nucleus was stained with DAPI for 5 min at room temperature. The positive signals were analysed using a confocal fluorescence microscope (Zeiss LSM510 Confocal).

### Determination of myeloperoxidase (MPO) level

The lung MPO level was determined using a commercial mouse MPO ELISA kit (Hycult Biotech) according to the manufacturer’s instructions.

### Statistical analysis

Data were displayed as mean ± SEM. Statistical analysis was performed using GraphPad Prism 5 program. Differences between groups were compared using the Student *t*-test or one-way analysis of variance (ANOVA). Statistically significant differences were considered as *P* < 0.05.

## Results

### WISP1 is highly increased in lung tissue following hepatic IRI

**Fig. 1:**
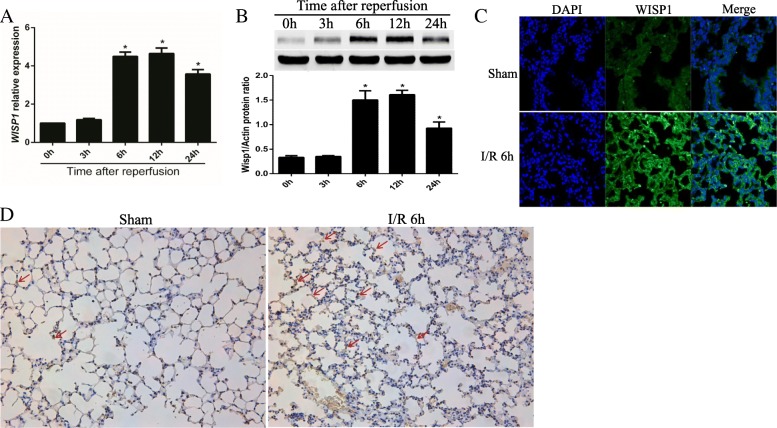
WISP1 is Highly Increased in Lung Tissue Following Hepatic IRI. qRT-PCR (**a**) and western blot (**b**) analysis of WISP1 in lung samples from WT mice undergoing ischemia for 60 min and reperfusion (I/R) for 0 h, 6 h, 12 h, and 24 h (*n* = 6 respectively). **c** Immunofluorescence and (**d**) Immunohistochemistry assays of WISP1 in lung samples from WT mice in sham and IRI group undergoing ischemia for 60 min and reperfusion for 6 h. Gene expression was normalized to *β-actin* mRNA levels in each sample. ^*^p < 0.05 compared with 0 h of reperfusion, data are expressed as mean ± s.e.m. for all samples

Previous work in our group has demonstrated WISP1 facilitates proinflammatory cascades following hepatic IRI in the liver dependent on TLR4 signaling [24]. Since remote organ injuries, such as lung injury [8], have been reported following hepatic IRI, we next sought to detect whether WISP1 was involved in the pathogenesis of lung injury in hepatic IRI. Interestingly, WISP1 gene transcription was found to be highly increased in the lung tissue following hepatic IRI after reperfusion for 6 h, 12 h, and 24 h (Fig. 1a). Western blot analysis further confirmed WISP1 protein level was also significantly upregulated (Fig. 1b). To further analyze WISP1 expression in morphology, we also performed immunofluorescence and Immunohistochemistry assays to verify WISP1 expression. Consistent with previous results, WISP1 is a secreted extracellular matrix protein mainly expressed in cytoplasm (Fig. 1c and d). WISP1 was highly expressed in cytoplasm of the cells in lung tissue (Fig. 1c and d). Together, these results indicated that WISP1 was highly increased in lung tissue following hepatic IRI.

### Treatment with anti-WISP1 antibody ameliorates lung injury following hepatic IRI

**Fig. 2:**
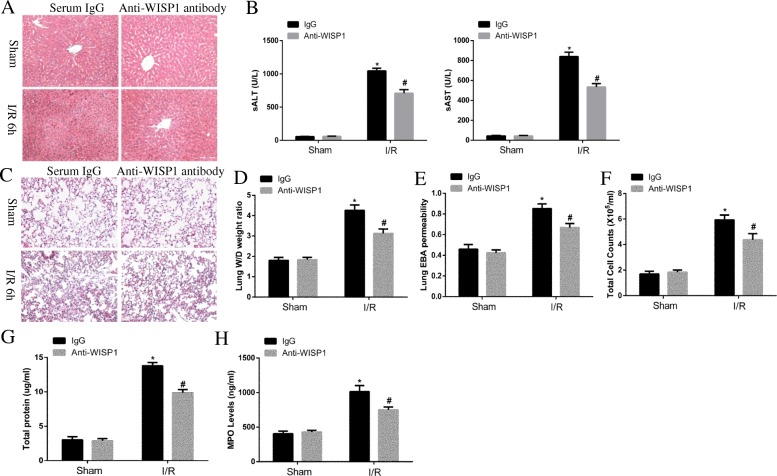
Treatment with Anti-WISP1 Antibody Ameliorates Lung Injury Following Hepatic IRI. WT mice in sham and IRI group undergoing ischemia for 60 min and reperfusion for 6 h were treated intraperitoneally with either control IgG antibody or anti-WISP1 antibody (6 μg/g) 1 h before ischemia and at the onset of reperfusion (*n* = 6 respectively). H&E staining (**a** and **c**), ALT and AST levels (**b**), W/D weight ratio (**d**), EBA permeability analysis (**e**), total cell counts (**f**) and proteins (**g**) in BALF and MPO levels (**h**) of lung samples from WT mice in sham and IRI group were performed. ^*^p < 0.05 compared with sham group, ^**#**^*p* < 0.05 compared with IgG group, data are expressed as mean ± s.e.m. for all samples

To further confirm the role of WISP1 in the pathogenesis of lung injury after hepatic IRI, we treated mice in sham and hepatic IRI group with neutralizing anti-WISP1 or control IgG antibody intraperitoneally 1 h before ischemia and again at the onset of reperfusion. After reperfusion for 6 h, mice were sacrificed and lung injury was assessed. Histopathology evaluation indicated treatment with anti-WISP1 antibody ameliorated liver injury, as showed by histopathological and biochemical (ALT/AST) evidence (Fig. 2a and b) and lung injury characterized by less diffuse interstitial edema and inflammatory cell infiltration (Fig. 2c), decreased W/D weight ratio (Fig. 2d) as well as decreased lung EBA permeability (Fig. 2e). Consistent with histopathology analysis, total cell counts (Fig. 2f) and total protein levels (Fig. 2g) were also decreased, indicating less severe inflammation compared with control group. MPO is a peroxidase enzyme which is abundantly expressed on polymorphonuclear neutrophils. The function of MPO has been associated with production of hypochlorous acid (HOCl) which is a subtype of ROS [27]. Consistently, we found MPO levels were also decreased in the lung tissue after anti-WISP1 antibody treatment following hepatic IRI (Fig. 2h). These results indicated that anti-WISP1 neutralizing antibody markedly ameliorated lung injury following hepatic IRI, further confirming the pathological role of WISP1.

### Treatment with anti-WISP1 antibody alters cytokine profiles in lung tissue following hepatic IRI

**Fig. 3:**
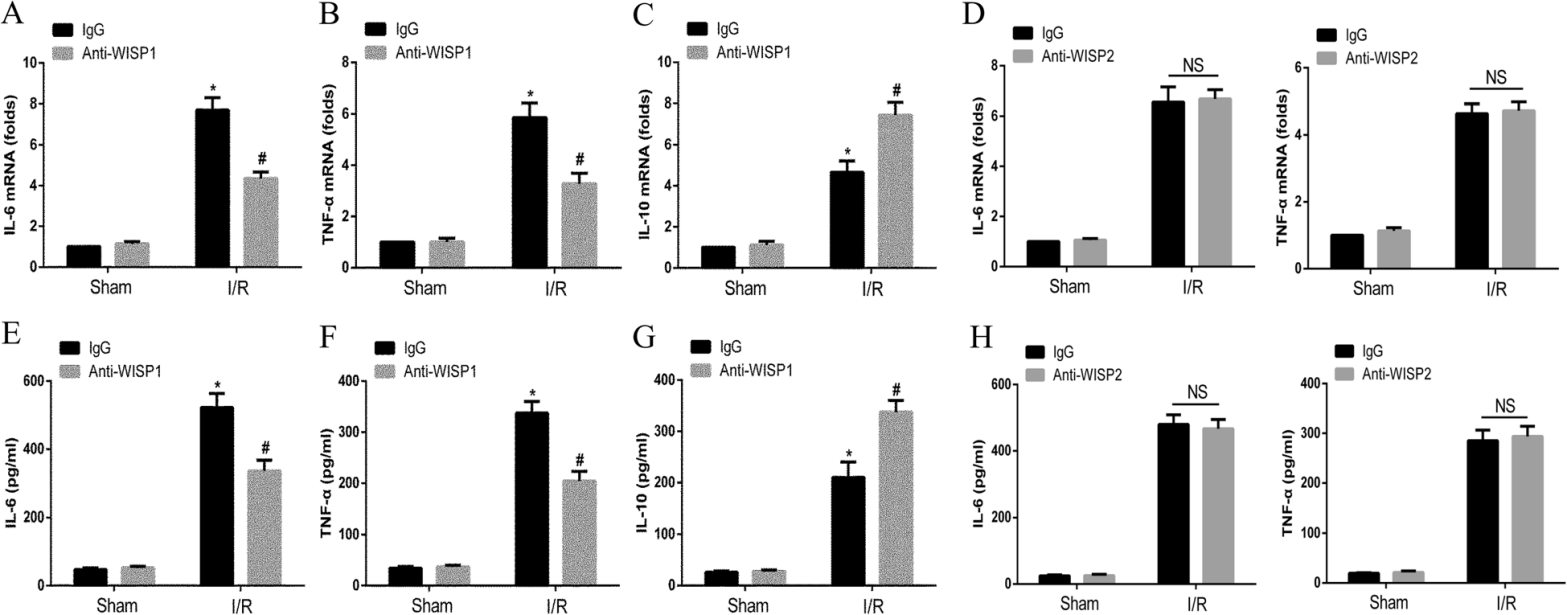
WISP1 facilitates the expression of pro-inflammatory cytokines in IBD LPMCs. WT mice in sham and IRI group undergoing ischemia for 60 min and reperfusion for 6 h were treated intraperitoneally with either control IgG antibody or neutralizing WISP1/WISP2 antibody (6 μg/g) 1 h before ischemia and at the onset of reperfusion (*n* = 6 respectively). qRT-PCR for IL-6 (**a** and **d**), TNF-α (**b** and **d**), and IL-10 (**c**) from lung tissues in all groups was performed. ELISA for IL-6 (**e** and **h**), TNF-α (**f** and **h**), and IL-10 (**g**) from BALF supernatants in all groups was performed. ^*^p < 0.05 compared with sham group, ^**#**^*p* < 0.05 compared with IgG group, data are expressed as mean ± s.e.m. for all samples

Previous results indicated anti-WISP1 antibody ameliorated lung injury following hepatic IRI. We next sought in determine whether treatment with anti-WISP1 antibody augmented proinflammatory or anti-inflammatory cytokine profiles which contributed to the injury in the lung tissue. Mice were treated as previously described. qRT-PCR analysis demonstrated that proinflammatory cytokine IL-6 (Fig. 3a) and TNF-α (Fig. 3b) transcriptions were significantly downregulated in lung tissue following hepatic IRI. However, anti-inflammatory cytokine IL-10 (Fig. 3c) was markedly upregulated. To further determine such cytokine alterations in protein level, we next performed ELISA analysis for these cytokines. In accordance with qRT-PCR results, IL-6 (Fig. 3e) and TNF-α (Fig. 3f) protein level were highly elevated and IL-10 (Fig. 3g) level was decreased. Additionally, we used anti-WISP2 antibody as a control to detect whether it has the same effect. There were no significant changes in pro-inflammatory cytokines’ transcription or release following neutralizing WISP2 antibody treatment during liver IRI (Fig. 3d and h). Together, these data suggested anti-WISP1 antibody led to decreased proinflammatory cytokine production and excessive anti-inflammatory cytokine secretion.

### Administration with rWISP1 aggravates lung injury following hepatic IRI

**Fig. 4:**
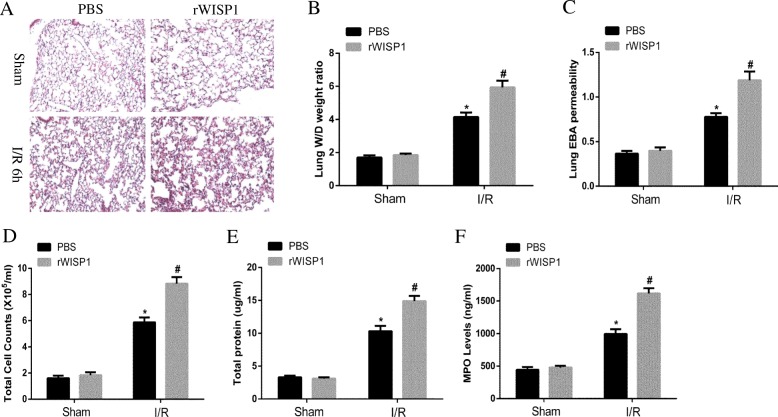
Administration with rWISP1 Aggravates Lung Injury Following Hepatic IRI. WT mice in sham and IRI group undergoing ischemia for 60 min and reperfusion for 6 h were treated intraperitoneally with either sterile PBS or rWISP1 (1 μg/g) at the onset of reperfusion (*n* = 6 respectively). H&E staining (**a**), W/D weight ratio (**b**), EBA permeability analysis (**c**), total cell counts (**d**) and proteins (**e**) in BALF and MPO levels (**f**) of lung samples from WT mice in sham and IRI group were performed.^*^p < 0.05 compared with sham group, ^**#**^*p* < 0.05 compared with PBS group, data are expressed as mean ± s.e.m. for all samples

To comprehensively study the pathological role of WISP1, mice in the sham and hepatic IRI group were administered with rWISP1 or control medium (sterile PBS) intraperitoneally once immediately after reperfusion. Lung injury was assessed as previously reported. Histopathology analysis suggested administration with rWISP1 aggravated lung injury characterized by more severe diffuse interstitial edema and more inflammatory cell infiltration (Fig. 4a), increased W/D weight ratio (Fig. 4b) as well as decreased lung EBA permeability (Fig. 4c). Total cell counts (Fig. 4d) and total protein levels (Fig. 4e) were also decreased, indicating severer inflammation compared with control group. Besides, MPO levels were also increased (Fig. 4f). These data indicated administration with rWISP1 aggravated lung injury following hepatic IRI, which contrasts with treatment with anti-WISP1 antibody.

### rWISP1 facilitates Proinflammatory cytokine and inhibits anti-inflammatory cytokine production in lung tissue following hepatic IRI

**Fig. 5:**
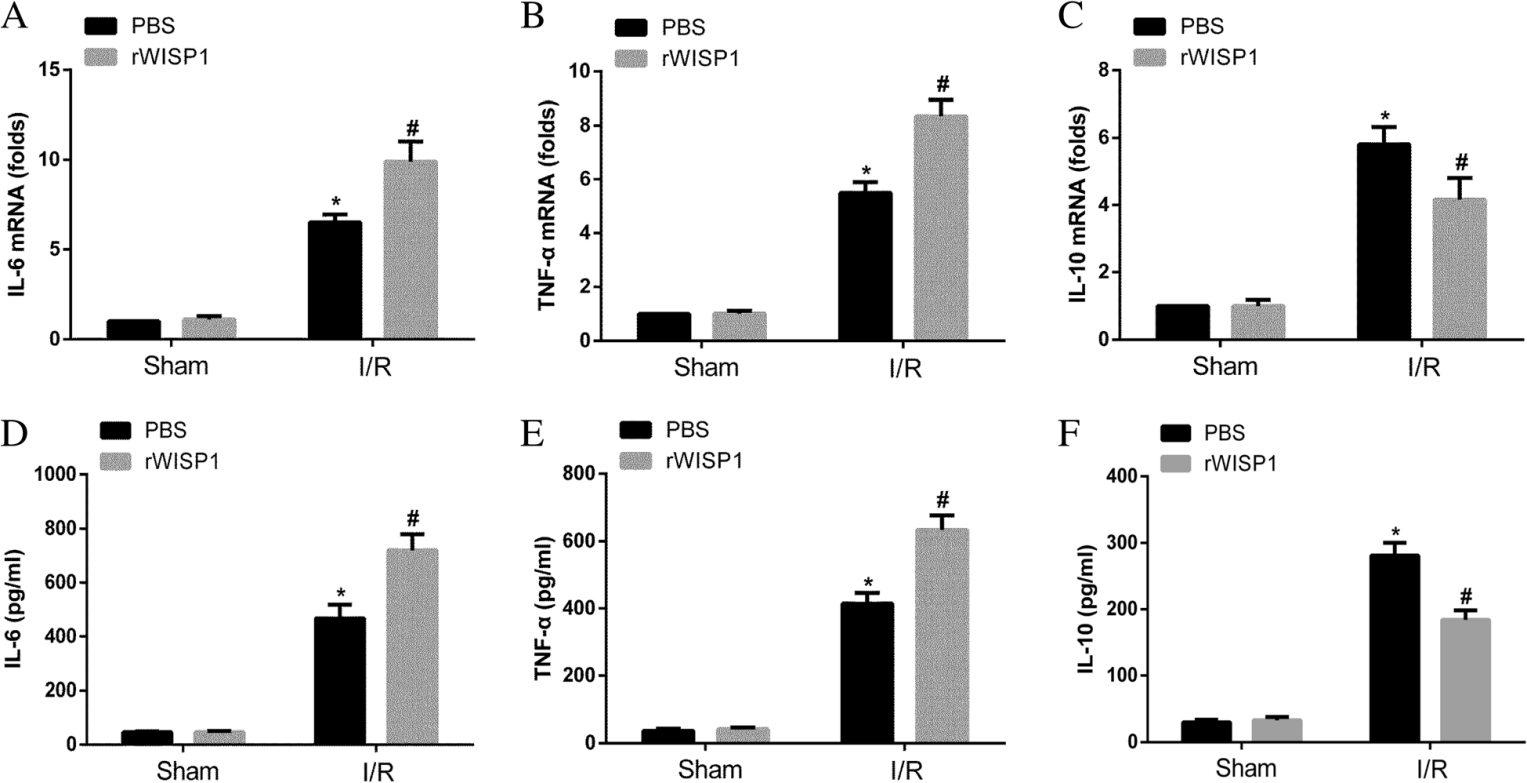
rWISP1 Facilitates Proinflammatory Cytokine and Inhibits Anti-inflammatory Cytokine Production in Lung Tissue Following Hepatic IRI. WT mice in sham and IRI group undergoing ischemia for 60 min and reperfusion for 6 h were treated intraperitoneally with either sterile PBS or rWISP1 (1 μg/g) at the onset of reperfusion (*n* = 6 respectively). qRT-PCR for IL-6 (**a**), TNF-α (**b**), and IL-10 (**c**) from lung tissues in all groups was performed. ELISA for IL-6 (**d**), TNF-α (**e**), and IL-10 (**f**) from BALF supernatants in all groups was performed.^*^p < 0.05 compared with sham group, ^**#**^*p* < 0.05 compared with PBS group, data are expressed as mean ± s.e.m. for all samples

Administration with rWISP1 significantly aggravated lung injury following hepatic IRI, we next wondered whether rWISP1 could alter inflammatory cytokine production in the lung tissue. Mice were treated as previously described. Lung tissues were harvested, and then qRT-PCR and ELISA analysis were performed. Consistently, qRT-PCR analysis suggested IL-6 (Fig. 5a) and TNF-α (Fig. 5b) transcript levels were significantly upregulated in lung tissue following hepatic IRI. IL-10 (Fig. 5c) was markedly downregulated. ELISA evaluation in protein level further confirmed the increased levels of IL-6 (Fig. 5d) and TNF-α (Fig. 5e) and decreased levels of IL-10 (Fig. 5f). Collectively, these results demonstrated administration with rWISP1 significantly facilitated proinflammatory cytokine and inhibited anti-inflammatory cytokine production in the lung after hepatic IRI, further validating the pathological role of WISP1.

### WISP1 contributes to lung injury in hepatic IRI dependent on TLR4

**Fig. 6:**
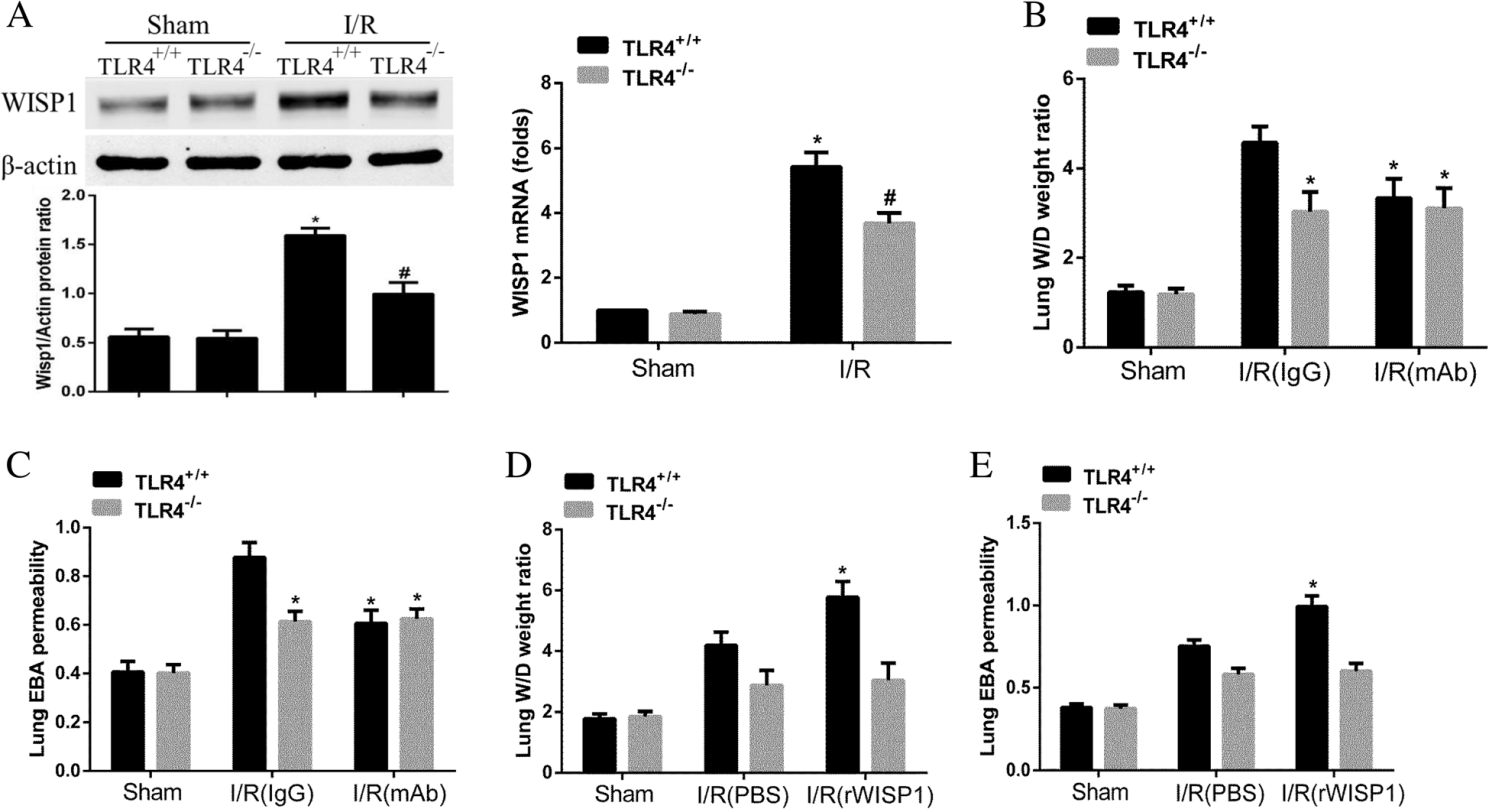
WISP1 Contributes to Lung Injury in Hepatic IRI Dependent on TLR4. WT and TLR4^−/−^ mice in sham and IRI group undergoing ischemia for 60 min and reperfusion for 6 h were treated intraperitoneally with either control IgG antibody or anti-WISP1 antibody (6 μg/g) 1 h before ischemia and at the onset of reperfusion (*n* = 6 respectively). In another experiment, WT and TLR4^−/−^ mice were treated intraperitoneally with either sterile PBS or rWISP1 (1 μg/g) at the onset of reperfusion (*n* = 6 respectively). Western blot and qRT-PCR analysis (**a**) of WISP1 in the lung tissue from all groups were performed. W/D weight ratio (**b**), EBA permeability (**c**) for mice treated with control IgG antibody or anti-WISP1 antibody were analyzed. W/D weight ratio (**d**) and EBA permeability (**e**) for mice administered with sterile PBS or rWISP1 were analyzed. **a** ^*^p < 0.05 compared with sham group, ^**#**^*p* < 0.05 compared with I/R(TLR4^+/+^) group, (**b**, **c**) ^*^p < 0.05 compared with I/R (IgG) group, (**d**, **e**) ^*^p < 0.05 compared with I/R (PBS) group, data are expressed as mean ± s.e.m. for all samples

Previous work has demonstrated WISP1 ameliorated lung injury in hepatic IRI through increased production of proinflammatory cytokines and inhibition of anti-inflammatory cytokines. We next sought to determine the specific mechanism of WISP1 in regulating inflammatory cascades in the lung. Previous studies have demonstrated that WISP1 interacted with TLR4 and contributed to ventilator-induced lung injury (VILI) dependent on TLR4 signaling [18]. However, the underlying mechanism of WISP1 in mediating lung injury following hepatic IRI still remains unknown. C57BL/6 WT (TLR^+/+^ in the Fig. 6) and TLR4^−/−^ mice were adopted in the experiment. Both mice were treated with control and IRI operation to determine the expression of WISP1. Interestingly, in IRI (I/R in the Fig. 6) group, WISP1 transcripts (Fig. 6a) and protein expression (Fig. 6a) in TLR4^−/−^ mice was decreased significantly compared with WT mice, indicating WISP1 expression in the lung tissue following hepatic IRI was dependent on TLR4. Next, we would like to examine whether WISP1 mediates inflammatory cascades through TLR4. WT and TLR4^−/−^ mice in sham and IRI group were treated with anti-WISP1 antibody and rWISP1 to evaluate lung injury. TLR4^−/−^ mice displayed decreased W/D ratio (Fig. 6b) and EBA permeability (Fig. 6c) compared with WT mice while no significance was detected between WT and TLR4^−/−^ mice in IRI group treated with anti-WISP1 antibody. However, W/D ratio (Fig. 6d) and EBA permeability (Fig. 6e) of TLR4^−/−^ mice in IRI group administered with rWISP1 were significantly decreased compared with those in WT mice, indicating rWISP1 could not induce lung injury in TLR4^−/−^ mice. Collectively, these date demonstrated WISP1 expression was dependent on TLR4 and WISP1 contributed to lung injury in hepatic IRI through TLR4.

## Discussion

Hepatic IRI is a common injury followed by liver transplantation, trauma, shock, etc. Besides liver injury, hepatic IRI also induce remote organ injuries, such as lung injury [8]. The underlying mechanisms of hepatic IRI still remain unclear, of which some mediators have been found playing indispensable roles in liver IRI induced lung injury, including high mobility group box-1 protein (HMGB1), adrenaline and N-acetyl-cysteine [28–30]. In the current study, we reported a new mediator, WISP1, plays a pivotal effect on hepatic IRI induced lung injury.

WNT1 inducible signaling pathway protein 1 (WISP1), one of the most important members in the CCN family, is a secreted extracellular matrix (ECM) which is ubiquitously expressed in various organs and tissues. The molecular function of CCN family has been associated with wound healing, organ fibrosis, cell survival and proliferation [31, 32]. WISP1 has been identified to be involved in the onset of several malignant diseases, such as breast cancer, hepatocellular carcinoma, colon adenocarcinomas, and lung carcinoma, osteoarthritis and lung fibrosis [16, 17, 33, 34]. In addition, we have demonstrated that WISP1 interacts with TLR4 by co-immunoprecipitation and mediates ventilator-induced lung injury (VILI) dependent on TLR4 signaling [18]. We also find WISP1 might contribute to hepatic ischemia reperfusion injury in mice and possibly depends on TLR4/TRIF signaling [24]. However, the role of WISP1 in the lung injury after IRI remains unknown.

This study aims to confirm the role of WISP1 in the lung injury following hepatic IRI and explore the possible regulating mechanisms. Interestingly, we found WISP1 transcript and protein expression were highly increased in the lung tissue following reperfusion for 6 h, 12 h and 24 h compared with sham mice. Immunofluorescence further confirmed WISP1 expression in the cytoplasm of cell in the lung. These results preliminarily indicated that WISP1 not only participated in the liver injury but also was involved in the pathogenesis of lung injury after hepatic IRI. To further validate the role of WISP1 in the lung injury, mice in the sham and IRI group were treated with anti-WISP1 antibody intraperitoneally to neutralize WISP1 in vivo. Fortunately, we found WISP1 neutralization significantly ameliorated lung injury after hepatic IRI compared to control IgG group characterized by less diffuse interstitial edema and inflammatory cell infiltration. Consistently, treatment with anti-WISP1 antibody markedly inhibited TNF-α and IL-6 production and enhanced IL-10 expression, further confirming the ameliorated lung injury. Conversely, administration with rWISP1 aggravated lung injury together with excessive expression of TNF-α and IL-6 as well as inhibition of IL-10 production. Since the expression and function of WISP1 has been closely linked to TLR4 pathway, we next sought to explore the potential relationship between WISP1 and TLR4. Interestingly, we found TLR4^−/−^ mice in IRI group displayed less transcript and protein expression of WISP1 compared with WT mice, preliminarily suggesting that WISP1 expression in lung injury after hepatic IRI was dependent on TLR4. Besides, rWISP1 could not induce lung injury in TLR4^−/−^ mice of IRI group compared with WT mice, further indicating that WISP1-mediated lung injury and inflammatory cascades might also dependent on TLR4. These data suggested there might be a circulatory regulating mechanism between WISP1 and TLR4 in the lung injury following hepatic IRI. However, the specific mechanisms how TLR4 regulates WISP1 expression and how WISP1 mediates inflammatory cascades through TLR4 need to be further elucidated in future studies.

In conclusion, our study demonstrated that WISP1 expression is upregulated in lung tissue following hepatic IRI. Anti-WISP1 antibody ameliorated lung injury with alterations in cytokine profiles. rWISP1 aggravated lung injury through excessive proinflammatory cytokine production and inhibition of anti-inflammatory cytokines. WISP1 contributes to lung injury through TLR4 pathway after hepatic IRI. Medications targeting WISP1 might be a promising approach for patients with lung injury following hepatic IRI.

## Abbreviations

BALF: Bronchoalveolar Lavage Fluid
ECM: Extracellular matrix
HMGB1: High mobility group box-1 protein
iNOS: Inducible nitric oxide synthase
IRI: Ischemia-reperfusion injury
MPO: Myeloperoxidase
PRRs: Pattern recognition receptors
ROS: reactive oxygen species
TLR4: Toll-like receptor 4
VILI: Ventilator-induced lung injury
WISP1: WNT1 inducible signaling pathway protein 1
